# Biphenyl-3,3′,4,4′-tetra­carboxylic acid dihydrate

**DOI:** 10.1107/S1600536808044012

**Published:** 2009-01-08

**Authors:** Fei Li, Wu-Wei Wang, Xing Ji, Chang-Chun Cao, Dong-Ya Zhu

**Affiliations:** a School of Pharmaceutical Science, Nanjing Medical University, Nanjing 210029, People’s Republic of China

## Abstract

The asymmetric unit of the title compound, C_16_H_10_O_8_·2H_2_O, contains one-half of the centrosymmetric organic mol­ecule and one water mol­ecule. The dihedral angles between the carboxyl­ate groups and the adjacent phenyl ring are 71.31 (3) and 16.67 (3)°, while the carboxyl­ate groups are oriented at a dihedral angle of 72.01 (3)°. In the crystal structure, inter­molecular O—H⋯O and bifurcated O—H⋯(O,O) hydrogen bonds link the mol­ecules to form a three-dimensional supra­molecular network.

## Related literature

For general background, see: Du *et al.* (2006 ▶, 2007 ▶); Desiraju (2003 ▶); Yaghi *et al.* (2003 ▶); Li *et al.* (2008 ▶). For a related structure, see: Coles *et al.* (2002 ▶). For bond-length data, see: Allen *et al.* (1987 ▶).
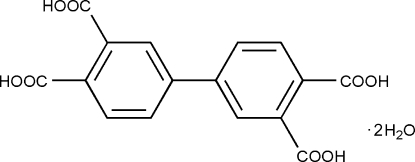


## Experimental

### 

#### Crystal data

C_16_H_10_O_8_·2H_2_O
*M* *_r_* = 366.27
Triclinic, 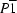

*a* = 5.5858 (16) Å
*b* = 6.6618 (19) Å
*c* = 11.086 (3) Å
α = 93.126 (5)°
β = 91.404 (4)°
γ = 109.110 (4)°
*V* = 388.81 (19) Å^3^
*Z* = 1
Mo *K*α radiation
μ = 0.13 mm^−1^
*T* = 296 (2) K
0.28 × 0.24 × 0.22 mm

#### Data collection

Bruker SMART CCD area-detector diffractometer
Absorption correction: multi-scan (*SADABS*; Bruker, 2001 ▶) *T*_min_ = 0.943, *T*_max_ = 0.973
1992 measured reflections
1362 independent reflections
1222 reflections with *I* > 2σ(*I*)
*R*_int_ = 0.008

#### Refinement

*R*[*F* ^2^ > 2σ(*F* ^2^)] = 0.033
*wR*(*F* ^2^) = 0.090
*S* = 1.08
1362 reflections
120 parameters
H-atom parameters constrained
Δρ_max_ = 0.16 e Å^−3^
Δρ_min_ = −0.16 e Å^−3^

### 

Data collection: *SMART* (Bruker, 2001 ▶); cell refinement: *SAINT* (Bruker, 2001 ▶); data reduction: *SAINT*; program(s) used to solve structure: *SHELXS97* (Sheldrick, 2008 ▶); program(s) used to refine structure: *SHELXL97* (Sheldrick, 2008 ▶); molecular graphics: *SHELXTL* (Sheldrick, 2008 ▶) and *ORTEP-3 for Windows* (Farrugia, 1997 ▶); software used to prepare material for publication: *SHELXTL*.

## Supplementary Material

Crystal structure: contains datablocks I, global. DOI: 10.1107/S1600536808044012/hk2568sup1.cif

Structure factors: contains datablocks I. DOI: 10.1107/S1600536808044012/hk2568Isup2.hkl

Additional supplementary materials:  crystallographic information; 3D view; checkCIF report

## Figures and Tables

**Table 1:** Hydrogen-bond geometry (Å, °)

*D*—H⋯*A*	*D*—H	H⋯*A*	*D*⋯*A*	*D*—H⋯*A*
O1—H1⋯O3^i^	0.82	1.88	2.683 (3)	168
O4—H4⋯O5^ii^	0.82	1.79	2.599 (3)	169
O5—H5*A*⋯O3^iii^	0.85	2.45	3.129 (3)	137
O5—H5*A*⋯O2^iv^	0.85	2.22	2.892 (3)	136
O5—H5*B*⋯O2	0.85	1.95	2.801 (3)	175

Symmetry codes: (i) 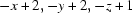; (ii) 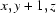; (iii) 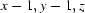; (iv) 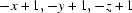.

**Fig. 1.**
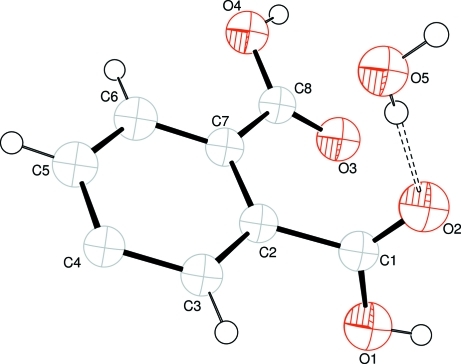
The asymmetric unit of the title molecule with the atom-numbering scheme. Hydrogen bond is shown as dashed line.

**Fig. 2.**
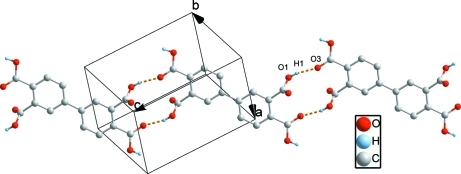
The one dimensional hydrogen bonded chain showing hydrogen bonds between the carboxyl units. Other H atoms have been omitted for clarity.

**Fig. 3.**
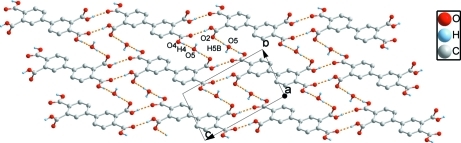
The two dimensional hydrogen-bonding layered structure along [011] direction.

**Fig. 4.**
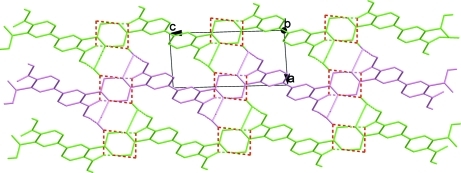
The three dimensional hydrogen-bonded supramolecular network. The adjacent layers are shown in green and rose colors, and interlayer hydrogen bonds are shown as red dashed lines.

## supplementary crystallographic information

### Comment

Non-covalent intermolecular interactions, mainly hydrogen bonding and aromatic stacking, play the key role to perfectly project and regulate the detailed crystal packing of supramolecular materials (Du *et al.*,  2006; Desiraju, 2003). Aromatic carboxylates have also been proved to be effective building blocks in constructing various architectures (Yaghi *et al.*,  2003; Li *et al.*,  2008; Du *et al.*,  2007). However, the crystal structures of these polycarboxyl acids themselves are rarely reported (Coles *et al.*,  2002). We synthesized the title compound under hydrothermal condition, and report herein its crystal structure.

The asymmetric unit of the title compound (Fig. 1) contains one-half of the centrosymmetric molecule and one water molecule. The bond lengths (Allen *et al.*,  1987) and angles are within normal ranges. The intramolecular O—H···O hydrogen bonding (Table 1) of the carboxylate O2 atom to the water molecule may cause to a small difference in the C1═O2 [1.2031 (18) Å] and C8═O3 [1.2156 (17) Å] double bonds of the carboxylate groups. The dihedral angles between the planar carboxylate groups (O1/C1/O2) and (O3/C8/O4) and the adjacent phenyl ring A (C2–C7) are 71.31 (3)° and 16.67 (3)°, respectively, while the carboxylate groups are oriented at a dihedral angle of 72.01 (3)°.

In the crystal structure, intra- and intermolecular O—H···O hydrogen bonds (Table 1) link the molecules to form a 3-D supramolecular network. Firstly, the O1—H1···O3 hydrogen bonds between the carboxyl units connect them into a 1-D zigzag chain (Fig. 2). Then, water molecules play the acceptor and donor roles, respectively, to participate in the formation of O4—H4···O5 and O5—H5B···O2 hydrogen bonds, giving rise to a 2-D supramolecular layer (Fig. 3). Finally, water molecules further act as donors to interconnect the supramolecular layers into 3-D networks with O5—H5A···O3 and O5—H5A···O2 hydrogen bonds (Fig. 4).

### Experimental

The title compound was recrystallized from the mixture of H_2_O (15 ml) and HNO_3_ (0.5 ml) under the hydrothermal conditions on cooling from 393 K. Colorless block shaped crystals were obtained at room temperature.

### Refinement

H atoms were positioned geometrically, with O—H = 0.82 Å (for OH), 0.85 Å (for OH_2_) and C—H = 0.93 Å for aromatic H, respectively, and constrained to ride on their parent atoms with U_iso_(H) = xU_eq_(C,O), where x = 1.2 for aromatic H and x = 1.5 for all other H atoms.

### Crystal data

**Table:**

C_16_H_10_O_8_·2H_2_O	*Z* = 1
*M**_r_* = 366.27	*F*(000) = 190
Triclinic, *P*1	*D*_x_ = 1.564 Mg m^−^^3^
Hall symbol: -P 1	Mo *K*α radiation, λ = 0.71073 Å
*a* = 5.5858 (16) Å	Cell parameters from 1245 reflections
*b* = 6.6618 (19) Å	θ = 3.2–27.8°
*c* = 11.086 (3) Å	µ = 0.13 mm^−^^1^
α = 93.126 (5)°	*T* = 296 K
β = 91.404 (4)°	Block, colourless
γ = 109.110 (4)°	0.28 × 0.24 × 0.22 mm
*V* = 388.81 (19) Å^3^	

### Data collection

**Table:**

Bruker SMART CCD area-detector diffractometer	1362 independent reflections
Radiation source: fine-focus sealed tube	1222 reflections with *I* > 2σ(*I*)
graphite	*R*_int_ = 0.008
φ and ω scans	θ_max_ = 25.0°, θ_min_ = 1.8°
Absorption correction: multi-scan (*SADABS*; Bruker, 2001)	*h* = −6→6
*T*_min_ = 0.943, *T*_max_ = 0.973	*k* = −7→7
1992 measured reflections	*l* = −9→13

### Refinement

**Table:**

Refinement on *F*^2^	Primary atom site location: structure-invariant direct methods
Least-squares matrix: full	Secondary atom site location: difference Fourier map
*R*[*F*^2^ > 2σ(*F*^2^)] = 0.033	Hydrogen site location: inferred from neighbouring sites
*wR*(*F*^2^) = 0.090	H-atom parameters constrained
*S* = 1.08	*w* = 1/[σ^2^(*F*_o_^2^) + (0.0467*P*)^2^ + 0.0831*P*] where *P* = (*F*_o_^2^ + 2*F*_c_^2^)/3
1362 reflections	(Δ/σ)_max_ < 0.001
120 parameters	Δρ_max_ = 0.16 e Å^−^^3^
0 restraints	Δρ_min_ = −0.16 e Å^−^^3^

### Special details

**Table:**

Geometry. All e.s.d.'s (except the e.s.d. in the dihedral angle between two l.s. planes) are estimated using the full covariance matrix. The cell e.s.d.'s are taken into account individually in the estimation of e.s.d.'s in distances, angles and torsion angles; correlations between e.s.d.'s in cell parameters are only used when they are defined by crystal symmetry. An approximate (isotropic) treatment of cell e.s.d.'s is used for estimating e.s.d.'s involving l.s. planes.
Refinement. Refinement of *F*^2^ against ALL reflections. The weighted *R*-factor *wR* and goodness of fit *S* are based on *F*^2^, conventional *R*-factors *R* are based on *F*, with *F* set to zero for negative *F*^2^. The threshold expression of *F*^2^ > σ(*F*^2^) is used only for calculating *R*-factors(gt) *etc*. and is not relevant to the choice of reflections for refinement. *R*-factors based on *F*^2^ are statistically about twice as large as those based on *F*, and *R*- factors based on ALL data will be even larger.

### Fractional atomic coordinates and isotropic or equivalent isotropic displacement parameters (Å^2^)

**Table:**

	*x*	*y*	*z*	*U*_iso_*/*U*_eq_	
O1	1.2031 (2)	0.8456 (2)	0.40618 (9)	0.0535 (3)	
H1	1.2130	0.8530	0.4803	0.080*	
O2	0.7989 (2)	0.6702 (2)	0.43405 (9)	0.0528 (3)	
O3	0.8059 (2)	1.09274 (19)	0.35300 (9)	0.0501 (3)	
O4	0.5352 (2)	1.08299 (18)	0.20056 (9)	0.0473 (3)	
H4	0.4952	1.1658	0.2468	0.071*	
O5	0.3760 (2)	0.35552 (17)	0.32212 (8)	0.0433 (3)	
H5A	0.2608	0.2949	0.3695	0.065*	
H5B	0.4996	0.4558	0.3561	0.065*	
C1	0.9670 (3)	0.7526 (2)	0.36863 (12)	0.0355 (3)	
C2	0.9236 (2)	0.7450 (2)	0.23342 (11)	0.0327 (3)	
C3	1.0093 (3)	0.6076 (2)	0.16299 (12)	0.0352 (3)	
H3	1.1062	0.5354	0.1992	0.042*	
C4	0.9533 (3)	0.5744 (2)	0.03778 (11)	0.0329 (3)	
C5	0.8074 (3)	0.6846 (3)	−0.01240 (12)	0.0426 (4)	
H5	0.7650	0.6639	−0.0949	0.051*	
C6	0.7238 (3)	0.8241 (3)	0.05735 (12)	0.0423 (4)	
H6	0.6272	0.8963	0.0210	0.051*	
C7	0.7815 (3)	0.8586 (2)	0.18104 (11)	0.0341 (3)	
C8	0.7072 (3)	1.0210 (2)	0.25400 (12)	0.0349 (3)	

### Atomic displacement parameters (Å^2^)

**Table:**

	*U*^11^	*U*^22^	*U*^33^	*U*^12^	*U*^13^	*U*^23^
O1	0.0436 (6)	0.0853 (9)	0.0246 (5)	0.0135 (6)	−0.0053 (4)	−0.0055 (5)
O2	0.0502 (7)	0.0738 (8)	0.0232 (5)	0.0062 (6)	0.0020 (5)	−0.0023 (5)
O3	0.0567 (7)	0.0651 (7)	0.0316 (6)	0.0290 (6)	−0.0118 (5)	−0.0202 (5)
O4	0.0621 (7)	0.0599 (7)	0.0298 (5)	0.0363 (6)	−0.0068 (5)	−0.0100 (5)
O5	0.0480 (6)	0.0484 (6)	0.0323 (5)	0.0154 (5)	0.0033 (4)	−0.0044 (4)
C1	0.0423 (8)	0.0418 (8)	0.0231 (7)	0.0164 (6)	−0.0016 (6)	−0.0054 (6)
C2	0.0342 (7)	0.0396 (7)	0.0216 (6)	0.0095 (6)	−0.0006 (5)	−0.0030 (5)
C3	0.0408 (8)	0.0430 (8)	0.0240 (7)	0.0175 (6)	−0.0019 (5)	−0.0016 (5)
C4	0.0375 (7)	0.0372 (7)	0.0226 (6)	0.0114 (6)	−0.0003 (5)	−0.0032 (5)
C5	0.0589 (10)	0.0540 (9)	0.0202 (7)	0.0276 (8)	−0.0063 (6)	−0.0065 (6)
C6	0.0559 (9)	0.0521 (9)	0.0265 (7)	0.0297 (8)	−0.0055 (6)	−0.0043 (6)
C7	0.0373 (7)	0.0390 (7)	0.0244 (7)	0.0113 (6)	−0.0004 (5)	−0.0036 (6)
C8	0.0381 (7)	0.0403 (8)	0.0250 (7)	0.0120 (6)	0.0007 (6)	−0.0025 (6)

### Geometric parameters (Å, °)

**Table:**

O1—C1	1.3065 (18)	C2—C7	1.399 (2)
O1—H1	0.8200	C3—C4	1.4047 (19)
O2—C1	1.2031 (18)	C3—H3	0.9300
O3—C8	1.2156 (17)	C4—C5	1.388 (2)
O4—C8	1.3052 (17)	C4—C4^i^	1.492 (3)
O4—H4	0.8200	C5—C6	1.380 (2)
O5—H5A	0.8500	C5—H5	0.9300
O5—H5B	0.8502	C6—C7	1.3901 (19)
C1—C2	1.5080 (18)	C6—H6	0.9300
C2—C3	1.380 (2)	C7—C8	1.4874 (19)
			
C1—O1—H1	109.5	C3—C4—C4^i^	121.05 (15)
C8—O4—H4	109.5	C6—C5—C4	121.45 (13)
H5A—O5—H5B	114.3	C6—C5—H5	119.3
O2—C1—O1	123.81 (12)	C4—C5—H5	119.3
O2—C1—C2	122.09 (13)	C5—C6—C7	121.10 (14)
O1—C1—C2	114.00 (12)	C5—C6—H6	119.5
C3—C2—C7	120.53 (12)	C7—C6—H6	119.5
C3—C2—C1	117.84 (12)	C6—C7—C2	118.11 (13)
C7—C2—C1	121.39 (12)	C6—C7—C8	120.75 (13)
C2—C3—C4	121.34 (13)	C2—C7—C8	121.04 (12)
C2—C3—H3	119.3	O3—C8—O4	123.78 (13)
C4—C3—H3	119.3	O3—C8—C7	122.06 (13)
C5—C4—C3	117.43 (13)	O4—C8—C7	114.11 (11)
C5—C4—C4^i^	121.52 (14)		
			
O2—C1—C2—C3	−104.45 (17)	C5—C6—C7—C2	−1.1 (2)
O1—C1—C2—C3	72.09 (17)	C5—C6—C7—C8	175.44 (14)
O2—C1—C2—C7	70.0 (2)	C3—C2—C7—C6	1.8 (2)
O1—C1—C2—C7	−113.51 (16)	C1—C2—C7—C6	−172.41 (13)
C7—C2—C3—C4	−1.2 (2)	C3—C2—C7—C8	−174.64 (12)
C1—C2—C3—C4	173.25 (13)	C1—C2—C7—C8	11.1 (2)
C2—C3—C4—C5	−0.3 (2)	C6—C7—C8—O3	−161.23 (14)
C2—C3—C4—C4^i^	−179.98 (15)	C2—C7—C8—O3	15.2 (2)
C3—C4—C5—C6	1.1 (2)	C6—C7—C8—O4	16.55 (19)
C4^i^—C4—C5—C6	−179.21 (16)	C2—C7—C8—O4	−167.06 (13)
C4—C5—C6—C7	−0.4 (3)		

Symmetry codes: (i) −*x*+2, −*y*+1, −*z*.

### Hydrogen-bond geometry (Å, °)

**Table:**

*D*—H···*A*	*D*—H	H···*A*	*D*···*A*	*D*—H···*A*
O1—H1···O3^ii^	0.82	1.88	2.683 (3)	168
O4—H4···O5^iii^	0.82	1.79	2.599 (3)	169
O5—H5A···O3^iv^	0.85	2.45	3.129 (3)	137
O5—H5A···O2^v^	0.85	2.22	2.892 (3)	136
O5—H5B···O2	0.85	1.95	2.801 (3)	175

Symmetry codes: (ii) −*x*+2, −*y*+2, −*z*+1; (iii) *x*, *y*+1, *z*; (iv) *x*−1, *y*−1, *z*; (v) −*x*+1, −*y*+1, −*z*+1.
